# Crystal structure and Hirshfeld surface analysis of 5-oxo-7-phenyl-2-(phenyl­amino)-1*H*-[1,2,4]triazolo[1,5-*a*]pyridine-6,8-dicarbo­nitrile dimethyl sulfoxide monosolvate

**DOI:** 10.1107/S2056989023004383

**Published:** 2023-05-26

**Authors:** Farid N. Naghiyev, Victor N. Khrustalev, Huseyn M. Mamedov, Mehmet Akkurt, Ali N. Khalilov, Ajaya Bhattarai, İbrahim G. Mamedov

**Affiliations:** a Faculty of Chemistry, Baku State University, Z. Khalilov str. 23, Az, 1148, Baku, Azerbaijan; b Peoples’ Friendship University of Russia (RUDN University), Miklukho-Maklay St. 6, Moscow, 117198, Russian Federation; c N. D. Zelinsky Institute of Organic Chemistry RAS, Leninsky Prosp. 47, Moscow, 119991, Russian Federation; d Faculty of Physics, Baku State University, Z. Khalilov str. 23, Az, 1148 Baku, Azerbaijan; e Department of Physics, Faculty of Sciences, Erciyes University, 38039 Kayseri, Türkiye; f Department of Chemistry, M.M.A.M.C (Tribhuvan University) Biratnagar, Nepal; University of Neuchâtel, Switzerland

**Keywords:** crystal structure, [1,2,4]triazolo[1,5-*a*]pyridine, hydrogen bond, Hirshfeld surface analysis

## Abstract

In the title compound, C_20_H_12_N_6_O·C_2_H_6_OS, the [1,2,4]triazolo[1,5-*a*]pyridine ring system is almost planar and makes dihedral angles of 16.33 (7) and 46.80 (7)°, respectively, with the phenyl­amino and phenyl rings. In the crystal, mol­ecules are linked by inter­molecular N—H⋯O and C—H⋯O hydrogen bonds into chains along the *b-*axis direction through the dimethyl sulfoxide solvent mol­ecule, forming *C*(10)*R* ^2^_1_(6) motifs. These chains are connected *via* S—O⋯π inter­actions, π–π stacking inter­actions between the pyridine rings [centroid-to-centroid distance = 3.6662 (9) Å] and van der Waals inter­actions. A Hirshfeld surface analysis of the crystal structure indicates that the most important contributions to the crystal packing are from H⋯H (28.1%), C⋯H/H⋯C (27.2%), N⋯H/H⋯N (19.4%) and O⋯H/H⋯O (9.8%) inter­actions.

## Abstract 2

In the crystal, inter­molecular N—H⋯O and C—H⋯O hydrogen bonds connect mol­ecules into chains along the *b-*axis direction through the dimethyl sulfoxide solvent mol­ecule, forming *C*(10) 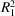 (6) motifs. These chains are connected *via* S—O⋯π inter­actions, π–π stacking inter­actions and van der Waals inter­actions.

## 1. Chemical context

Diverse carbon–carbon and carbon–heteroatom bond-formation reactions are considered fundamental tools in organic synthesis. The reaction has also been amplified, extending these methods to different fields of chemistry, as well to the synthesis of natural products, in medicinal and pharmaceutical chemistry, material science, supra­molecular chemistry *etc* (Çelik *et al.*, 2023 ▸; Chalkha *et al.*, 2023 ▸; Tapera *et al.*, 2022 ▸; Gurbanov *et al.*, 2020 ▸; Zubkov *et al.*, 2018 ▸). Triazolo[1,5-*a*]pyridines are accessible heterocyclic compounds and α-substituted pyridines are among the most widely used starting materials for their synthesis. The most common synthetic pathways to these compounds are well-reviewed in the literature (Jones & Abarca, 2010 ▸; Soliman *et al.*, 2014 ▸; Kotovshchikov *et al.*, 2021 ▸). The triazolo[1,5-*a*]pyridine moiety is a widespread structural motif in various synthetic biologically active compounds, possessing cardiovascular, trypanocidal, nitric oxide synthase inhibitor and anti­microbial activity, and in non-hormonal compounds with anti­fertility activity and leishmanicidal activity (Jones & Abarca, 2010 ▸; Mohamed *et al.*, 2013 ▸; Poustforoosh *et al.*, 2022 ▸).

**Figure 1:**
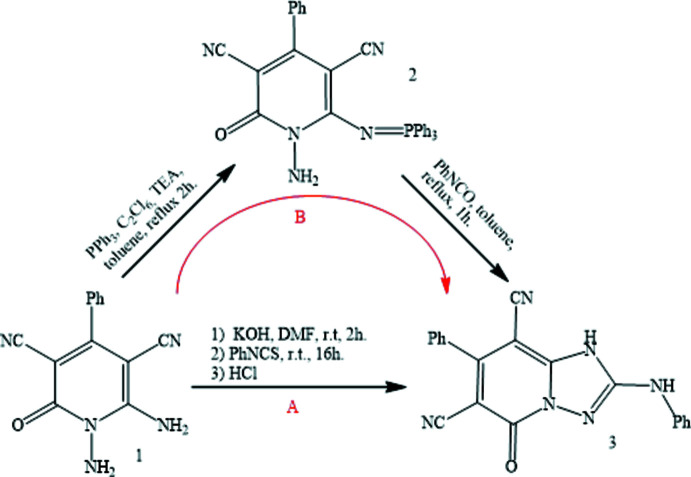
The synthesis routes or the title compound **3**.

A literature survey shows that the title compound **3** was previously synthesized in a two-pot reaction protocol (Barsy *et al.*, 2008 ▸), wherein the imino­phospho­rane 1-amino-6-(tri­phenyl­phospho­ranyl­idene­amino)-2-oxo-4-phenyl-1,2-di­hydro­pyri­dine-3,5-dicarbo­nitrile **2** prepared from 1,6-di­amino­pyridine **1** reacted with phenyl­iso­cyanate method to prepare **3** (*B* pathway, Fig. 1 ▸). Herein, we disclose a more straightforward one-pot synthesis method of **3** using the same starting compound **1** at room temperature (*A* pathway, Fig. 1 ▸), but through a different pathway. 
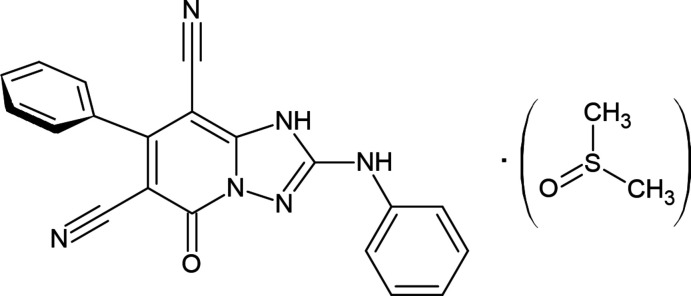


Continuing our investigations of heterocyclic systems with biological activity and in the framework of our ongoing structural studies (Maharramov *et al.*, 2021 ▸, 2022 ▸; Naghiyev *et al.*, 2020 ▸, 2021 ▸, 2022 ▸), we report the crystal structure and Hirshfeld surface analysis of the title compound, 5-oxo-7-phenyl-2-(phenyl­amino)-1,5-di­hydro-[1,2,4]triazolo[1,5-*a*]pyridine-6,8-dicarbo­nitrile, which crystallized as a DMSO solvate.

## 2. Structural commentary

**Figure 2:**
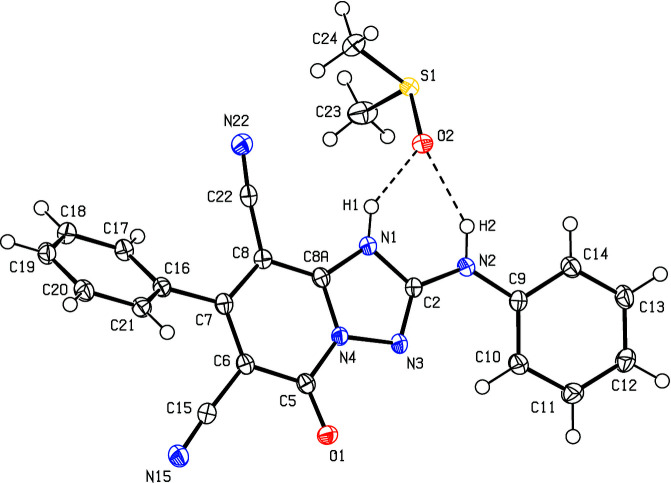
The mol­ecular structure of the title compound, showing the atom labelling and displacement ellipsoids drawn at the 50% probability level.

In the title compound, (Fig. 2 ▸), the [1,2,4]triazolo[1,5-*a*]pyridine ring system (N1/N3/N4/C2/C5–C8/C8*A*) is almost planar [maximum deviation = 0.043 (2) Å for C5] and subtends dihedral angles of 16.33 (7) and 46.80 (7)°, respectively, with the phenyl­amino and phenyl rings (C9–C14 and C16–C21). The geometric properties of the title compound are normal and consistent with those of the related compounds listed in the *Database survey* (Section 4).

## 3. Supra­molecular features

**Figure 3:**
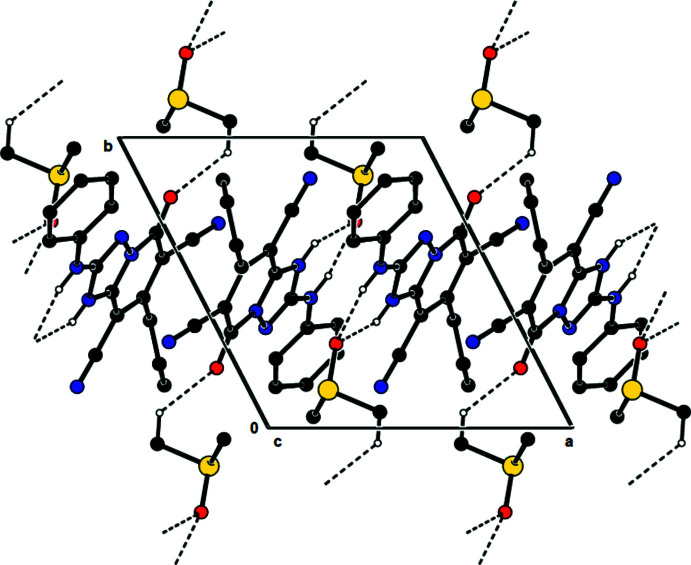
A view along the *c* axis of the N—H⋯O and C—H⋯O bonds in the title compound.

In the crystal, mol­ecules are linked by inter­molecular N—H⋯O and C—H⋯O hydrogen bonds into chains along the *b*-axis direction through the dimethyl sulfoxide solvent mol­ecule, forming *C*(10) 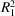 (6) motifs (Bernstein *et al.*, 1995 ▸; Table 1 ▸). These chains are connected *via* S—O⋯π inter­actions [S1—O2⋯*Cg*2^i^: O2⋯*Cg*2^i^ = 3.1775 (14) Å; S1⋯*Cg*2^i^ = 4.0054 (8) Å; S1—O2⋯*Cg*2^i^ = 111.93 (6)°; symmetry code: (i) 1 − *x*, 1 − *y*, 1 − *z*; *Cg*2 is the centroid of the pyridine ring (N4/C5–C8/C8*A*)], π–π stacking inter­actions [*Cg*2⋯*Cg*2^ii^ = 3.6662 (9) Å; slippage = 1.468 Å; symmetry code: (ii) −*x*, 1 − *y*, 1 − *z*] and van der Waals inter­actions (Fig. 3 ▸).

**Figure 4:**
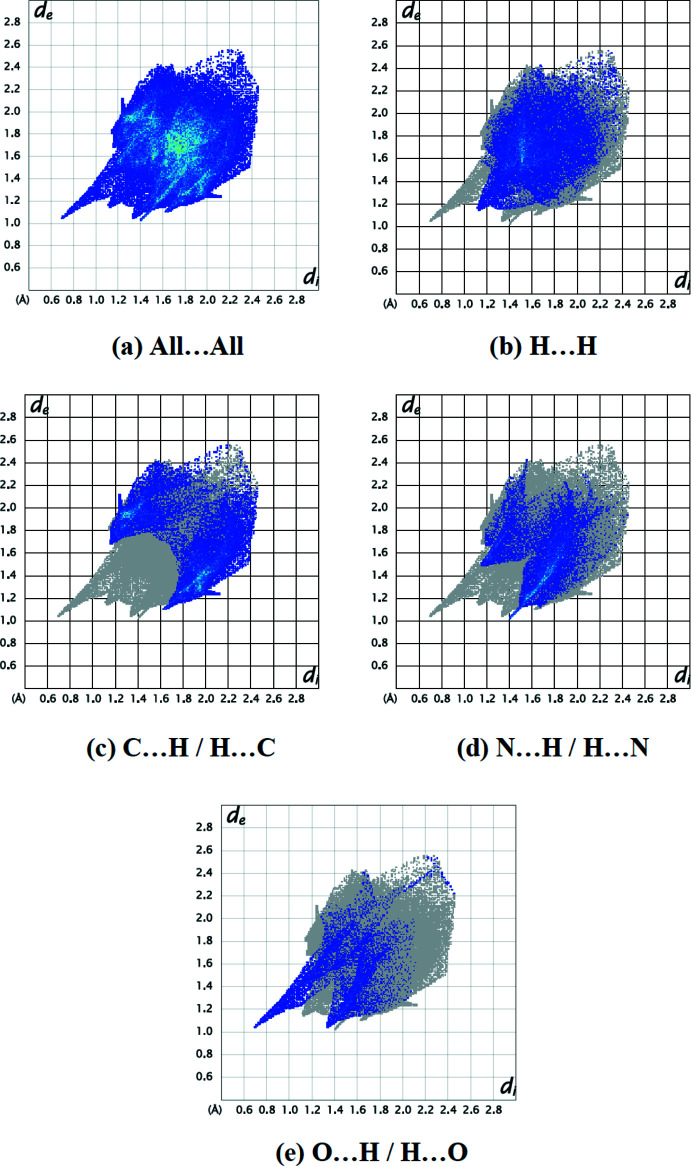
Two-dimensional fingerprint plots for title mol­ecule showing (*a*) all inter­actions, and delineated into (*b*) H⋯H, (*c*) C⋯H/H⋯C, (*d*) N⋯H/H⋯N and (*e*) O⋯H/H⋯O inter­actions. The *d*_i_ and *d*_e_ values are the closest inter­nal and external distances (in Å) from given points on the Hirshfeld surface.

*CrystalExplorer17.5* (Spackman *et al.*, 2021 ▸) was used to compute Hirshfeld surfaces of the title mol­ecule and two-dimensional fingerprints. The Hirshfeld surfaces were mapped over *d*_norm_ in the range −0.6769 (red) to +1.1190 (blue) a.u. The inter­actions given in Table 2 ▸ play a key role in the mol­ecular packing of the title compound. The most important inter­atomic contact is H⋯H as it makes the highest contribution to the crystal packing (28.1%, Fig. 4 ▸ *b*). Other major contributors are C⋯H/H⋯C (27.2%, Fig. 4 ▸ *c*), N⋯H/H⋯N (19.4%, Fig. 4 ▸ *d*) and N⋯H/H⋯N (9.8%, Fig. 4 ▸ *e*) inter­actions. Smaller contributions are made by N⋯C/C⋯N (6.7%), C⋯C (3.6%), O⋯C/C⋯O(1.7%), N⋯N (1.5%), S⋯H/H⋯S (1.0%), O⋯N/N⋯O (0.7%), S⋯C/C⋯S(0.2%) and O⋯S/S⋯O (0.1%) inter­actions.

## 4. Database survey

A search of the Cambridge Structural Database (CSD, Version 5.42, update of September 2021; Groom *et al.*, 2016 ▸) for the central nine-membered ring system ‘1,5-di­hydro­[1,2,4]triazolo[1,5-*a*]pyridine’ yielded three compounds related to the title compound, *viz*. CSD refcodes HODQEZ (Gumus *et al.*, 2019 ▸), HODQID (Gumus *et al.*, 2019 ▸) and RETCAX (Aydemir *et al.*, 2018 ▸).

In the crystal of HODQEZ, pairs of N—H⋯N hydrogen bonds link the mol­ecules, forming inversion dimers with an 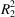 (8) ring motif. The dimers are linked by C—H⋯π and C—Br⋯π inter­actions, forming layers parallel to the *bc* plane. In the crystal of HODQID, mol­ecules are linked by N—H⋯N and C—H⋯O hydrogen bonds, forming chains propagating along the *b*-axis direction. In the crystal of RETCAX, N—H⋯N hydrogen bonds link the mol­ecules into supra­molecular chains propagating along the *c-*axis direction.

## 5. Synthesis and crystallization

To a solution of 1,6-di­amino-2-oxo-4-phenyl-1,2-di­hydro­pyridine-3,5-dicarbo­nitrile (0.82 g, 5.1 mmol) in DMF (25 mL) was added 10 mL of an aqueous solution of potassium hydroxide (0.28 g, 5.1 mmol). The mixture was stirred at room temperature for 2 h. Then an equimolar amount of phenyl­iso­thio­cyanate (0.51 g, 5.2 mmol) was added to the vigorously stirred reaction mixture and left overnight. After completion of the reaction, monitored by TLC, the reaction mixture was acidified by adding conc. HCl (4 mL). The precipitated solids were separated by filtration and recrystallized from an ethanol/water (1:1) solution (yield 80%; m.p. 557–558 K). Single crystals were grown from a DMSO solution.

^1^H NMR (300 MHz, DMSO-*d*_6_, p.p.m.): 4.3 (*s*, 1H, NH); 6.9 (*t*, 1H, CH_arom_, ^3^ *J*_H—H_ = 7.5 MHz); 7.3 (*t*, 2H, CH_arom_, ^3^ *J*_H—H_ = 7.5 MHz); 7.5 (*m*, 5H, CH_arom_); 7.7 (*d*, 2H, CH_arom_, ^3^ *J*_H—H_ = 8.1 MHz); 9.6 (*s*, 1H, NH); ^13^C NMR (75 MHz, DMSO-*d*_6_, p.p.m.): 76.4 (C_quat_), 83.9 (C_quat_), 117.0 (CH_arom_), 117.5 (CN), 118.9 (CN), 120.7 (CH_arom_), 128.9 (CH_arom_), 129.0 (CH_arom_), 129.2 (CH_arom_), 129.9 (CH_arom_), 136.3 (C_arom_), 141.4 (C_arom_), 152.2 (C_quat_), 154.9 (C_quat_), 156.2 (C_quat_), 161.1 (C=O).

## 6. Refinement

Crystal data, data collection and structure refinement details are summarized in Table 3 ▸. The NH H atoms were located in a difference-Fourier map [N1—H1 = 0.88 (2) Å and N2—H2 = 0.90 (2) Å] and refined with *U*_iso_(H) = 1.2*U*_eq_(N). Carbon-bound H atoms were positioned geometrically [C—H = 0.95–0.98 Å;] and were included in the refinement in the riding-model approximation with *U*_iso_(H) = 1.2 or 1.5*U*_eq_(C).

## Supplementary Material

Crystal structure: contains datablock(s) I. DOI: 10.1107/S2056989023004383/tx2068sup1.cif

Structure factors: contains datablock(s) I. DOI: 10.1107/S2056989023004383/tx2068Isup2.hkl

Supporting information file. DOI: 10.1107/S2056989023004383/tx2068Isup3.cml

CCDC reference: 2264500

Additional supporting information:  crystallographic information; 3D view; checkCIF report

## Figures and Tables

**Table 1:** Hydrogen-bond geometry (Å, °)

*D*—H⋯*A*	*D*—H	H⋯*A*	*D*⋯*A*	*D*—H⋯*A*
N1—H1⋯O2	0.88 (2)	1.84 (2)	2.6249 (16)	146.9 (17)
N2—H2⋯O2	0.90 (2)	2.07 (2)	2.8680 (16)	147.7 (17)
C10—H10⋯N3	0.95	2.39	2.9956 (19)	121
C23—H23*B*⋯O1^i^	0.98	2.44	3.166 (2)	131
C24—H24*B*⋯N22	0.98	2.53	3.474 (2)	162

Symmetry code: (i) 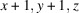 .

**Table 2:** Summary of short inter­atomic contacts (Å) in the title compound

Contact	Distance	Symmetry operation
O1⋯H23*B*	2.44	−1 + *x*, −1 + *y*, *z*
H17⋯O1	2.65	−*x*, 1 − *y*, 1 − *z*
O1⋯H24*A*	2.63	1 − *x*, 1 − *y*, 1 − *z*
H1⋯O2	1.84	*x*, *y*, *z*
H14⋯C21	2.98	1 − *x*, 1 − *y*, 1 − *z*
H19⋯N15	2.73	−*x*, 1 − *y*, 2 − *z*
H18⋯N22	2.82	1 − *x*, 2 − *y*, 2 − *z*
C22⋯H23*B*	3.08	1 − *x*, 2 − *y*, 1 − *z*
C9⋯H20	3.05	*x*, *y*, −1 + *z*
C11⋯C11	3.53	−*x*, −*y*, −*z*
C12⋯H18	3.05	*x*, −1 + *y*, −1 + *z*
H19⋯H23*A*	2.54	*x*, *y*, 1 + *z*
H12⋯H24*A*	2.41	−1 + *x*, −1 + *y*, −1 + *z*
H12⋯S1	3.04	1 − *x*, 1 − *y*, −*z*
H23*C*⋯H23*C*	2.43	1 − *x*, 2 − *y*, 1 − *z*

**Table 3:** Experimental details

Crystal data	
Chemical formula	C_20_H_12_N_6_O·C_2_H_6_OS
*M*_r_	430.48
Crystal system, space group	Triclinic, *P* 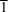
Temperature (K)	100
*a*, *b*, *c* (Å)	9.87885 (12), 10.46018 (13), 11.48307 (12)
α, β, γ (°)	100.9305 (10), 105.3054 (11), 112.6790 (12)
*V* (Å^3^)	997.81 (2)
*Z*	2
Radiation type	Cu *K*α
μ (mm^−1^)	1.73
Crystal size (mm)	0.22 × 0.16 × 0.12

Data collection	
Diffractometer	XtaLAB Synergy, Dualflex, HyPix
Absorption correction	Multi-scan (*CrysAlis PRO*; Rigaku OD, 2021 ▸)
*T*_min_, *T*_max_	0.660, 0.781
No. of measured, independent and observed [*I* > 2σ(*I*)] reflections	22299, 4314, 4124
*R*_int_	0.037
(sin θ/λ)_max_ (Å^−1^)	0.638

Refinement	
*R*[*F* ^2^ > 2σ(*F* ^2^)], *wR*(*F* ^2^), *S*	0.038, 0.106, 1.08
No. of reflections	4314
No. of parameters	289
H-atom treatment	H atoms treated by a mixture of independent and constrained refinement
Δρ_max_, Δρ_min_ (e Å^−3^)	0.41, −0.51

Computer programs: *CrysAlis PRO* (Rigaku OD, 2021 ▸), *SHELXT* (Sheldrick, 2015*a* ▸), *SHELXL* (Sheldrick, 2015*b* ▸), *ORTEP-3 for Windows* (Farrugia, 2012 ▸) and *PLATON* (Spek, 2020 ▸).

## supplementary crystallographic information

### Crystal data

**Table:**

C_20_H_12_N_6_O·C_2_H_6_OS	*Z* = 2
*M**_r_* = 430.48	*F*(000) = 448
Triclinic, *P*1	*D*_x_ = 1.433 Mg m^−^^3^
*a* = 9.87885 (12) Å	Cu *K*α radiation, λ = 1.54184 Å
*b* = 10.46018 (13) Å	Cell parameters from 15870 reflections
*c* = 11.48307 (12) Å	θ = 4.8–79.3°
α = 100.9305 (10)°	µ = 1.73 mm^−^^1^
β = 105.3054 (11)°	*T* = 100 K
γ = 112.6790 (12)°	Prism, colourless
*V* = 997.81 (2) Å^3^	0.22 × 0.16 × 0.12 mm

### Data collection

**Table:**

XtaLAB Synergy, Dualflex, HyPix diffractometer	4124 reflections with *I* > 2σ(*I*)
Radiation source: micro-focus sealed X-ray tube	*R*_int_ = 0.037
φ and ω scans	θ_max_ = 79.5°, θ_min_ = 4.2°
Absorption correction: multi-scan (CrysAlisPro; Rigaku OD, 2021)	*h* = −12→9
*T*_min_ = 0.660, *T*_max_ = 0.781	*k* = −12→13
22299 measured reflections	*l* = −14→14
4314 independent reflections	

### Refinement

**Table:**

Refinement on *F*^2^	Secondary atom site location: difference Fourier map
Least-squares matrix: full	Hydrogen site location: mixed
*R*[*F*^2^ > 2σ(*F*^2^)] = 0.038	H atoms treated by a mixture of independent and constrained refinement
*wR*(*F*^2^) = 0.106	*w* = 1/[σ^2^(*F*_o_^2^) + (0.0554*P*)^2^ + 0.554*P*] where *P* = (*F*_o_^2^ + 2*F*_c_^2^)/3
*S* = 1.08	(Δ/σ)_max_ < 0.001
4314 reflections	Δρ_max_ = 0.41 e Å^−^^3^
289 parameters	Δρ_min_ = −0.51 e Å^−^^3^
0 restraints	Extinction correction: SHELXL, Fc^*^=kFc[1+0.001xFc^2^λ^3^/sin(2θ)]^-1/4^
Primary atom site location: difference Fourier map	Extinction coefficient: 0.0029 (4)

### Special details

**Table:**

Experimental. CrysAlisPro 1.171.41.117a (Rigaku OD, 2021) Empirical absorption correction using spherical harmonics, implemented in SCALE3 ABSPACK scaling algorithm.
Geometry. All esds (except the esd in the dihedral angle between two l.s. planes) are estimated using the full covariance matrix. The cell esds are taken into account individually in the estimation of esds in distances, angles and torsion angles; correlations between esds in cell parameters are only used when they are defined by crystal symmetry. An approximate (isotropic) treatment of cell esds is used for estimating esds involving l.s. planes.

### Fractional atomic coordinates and isotropic or equivalent isotropic displacement parameters (Å^2^)

**Table:**

	*x*	*y*	*z*	*U*_iso_*/*U*_eq_	
O1	−0.06967 (12)	0.20611 (11)	0.42668 (10)	0.0214 (2)	
N1	0.37412 (14)	0.55891 (13)	0.44627 (11)	0.0171 (2)	
H1	0.465 (2)	0.635 (2)	0.4612 (18)	0.021*	
C2	0.29261 (16)	0.44403 (15)	0.33251 (13)	0.0163 (3)	
N2	0.35694 (14)	0.44663 (14)	0.24222 (12)	0.0189 (3)	
H2	0.453 (2)	0.523 (2)	0.2678 (18)	0.023*	
N3	0.15858 (14)	0.34368 (13)	0.32865 (11)	0.0178 (2)	
N4	0.15615 (14)	0.40235 (12)	0.44782 (11)	0.0159 (2)	
C5	0.03861 (16)	0.32893 (15)	0.49174 (14)	0.0177 (3)	
C6	0.06152 (16)	0.41513 (15)	0.61676 (13)	0.0176 (3)	
C7	0.19229 (16)	0.55162 (15)	0.69078 (13)	0.0174 (3)	
C8	0.30798 (16)	0.61215 (15)	0.63984 (13)	0.0172 (3)	
C8A	0.28510 (16)	0.53211 (15)	0.51784 (13)	0.0167 (3)	
C9	0.29501 (17)	0.34391 (15)	0.11906 (13)	0.0179 (3)	
C10	0.13802 (18)	0.23723 (17)	0.06025 (15)	0.0227 (3)	
H10	0.0659	0.2308	0.1023	0.027*	
C11	0.08828 (19)	0.13987 (18)	−0.06143 (15)	0.0262 (3)	
H11	−0.0189	0.0670	−0.1026	0.031*	
C12	0.19282 (19)	0.14769 (18)	−0.12345 (15)	0.0265 (3)	
H12	0.1581	0.0794	−0.2056	0.032*	
C13	0.34888 (19)	0.25654 (18)	−0.06421 (15)	0.0242 (3)	
H13	0.4208	0.2634	−0.1065	0.029*	
C14	0.39982 (17)	0.35488 (17)	0.05611 (14)	0.0211 (3)	
H14	0.5062	0.4298	0.0958	0.025*	
C15	−0.06863 (17)	0.35194 (15)	0.65539 (13)	0.0186 (3)	
N15	−0.18041 (15)	0.29648 (14)	0.67726 (13)	0.0235 (3)	
C16	0.21011 (16)	0.63048 (16)	0.82000 (13)	0.0178 (3)	
C17	0.25352 (17)	0.78044 (16)	0.85674 (14)	0.0202 (3)	
H17	0.2689	0.8319	0.7977	0.024*	
C18	0.27428 (18)	0.85436 (16)	0.97909 (14)	0.0223 (3)	
H18	0.3041	0.9563	1.0037	0.027*	
C19	0.25145 (17)	0.77930 (17)	1.06577 (14)	0.0218 (3)	
H19	0.2658	0.8301	1.1495	0.026*	
C20	0.20758 (17)	0.62989 (17)	1.03002 (14)	0.0215 (3)	
H20	0.1913	0.5788	1.0892	0.026*	
C21	0.18757 (17)	0.55538 (16)	0.90782 (14)	0.0195 (3)	
H21	0.1587	0.4537	0.8839	0.023*	
C22	0.44758 (17)	0.74971 (16)	0.70142 (14)	0.0191 (3)	
N22	0.56198 (16)	0.85887 (14)	0.74045 (13)	0.0251 (3)	
S1	0.73840 (4)	0.86862 (4)	0.42677 (3)	0.01894 (11)	
O2	0.63433 (12)	0.71002 (11)	0.41226 (10)	0.0215 (2)	
C23	0.6060 (2)	0.94489 (18)	0.39068 (19)	0.0328 (4)	
H23A	0.5447	0.9058	0.2985	0.049*	
H23B	0.6662	1.0518	0.4170	0.049*	
H23C	0.5340	0.9190	0.4366	0.049*	
C24	0.83449 (19)	0.96161 (17)	0.59410 (15)	0.0249 (3)	
H24A	0.9001	0.9187	0.6319	0.037*	
H24B	0.7551	0.9518	0.6326	0.037*	
H24C	0.9013	1.0657	0.6101	0.037*	

### Atomic displacement parameters (Å^2^)

**Table:**

	*U*^11^	*U*^22^	*U*^33^	*U*^12^	*U*^13^	*U*^23^
O1	0.0204 (5)	0.0187 (5)	0.0200 (5)	0.0048 (4)	0.0082 (4)	0.0039 (4)
N1	0.0170 (6)	0.0169 (5)	0.0174 (6)	0.0073 (5)	0.0079 (4)	0.0045 (4)
C2	0.0179 (6)	0.0170 (6)	0.0160 (6)	0.0096 (5)	0.0072 (5)	0.0054 (5)
N2	0.0173 (6)	0.0196 (6)	0.0181 (6)	0.0065 (5)	0.0089 (5)	0.0037 (5)
N3	0.0193 (6)	0.0192 (6)	0.0162 (6)	0.0087 (5)	0.0095 (5)	0.0049 (5)
N4	0.0174 (6)	0.0156 (5)	0.0157 (5)	0.0075 (5)	0.0080 (4)	0.0046 (4)
C5	0.0187 (6)	0.0193 (6)	0.0191 (7)	0.0108 (5)	0.0086 (5)	0.0083 (5)
C6	0.0195 (7)	0.0199 (7)	0.0177 (7)	0.0109 (6)	0.0091 (5)	0.0081 (5)
C7	0.0198 (7)	0.0198 (7)	0.0178 (7)	0.0128 (6)	0.0077 (5)	0.0080 (5)
C8	0.0181 (6)	0.0175 (6)	0.0172 (7)	0.0089 (5)	0.0073 (5)	0.0058 (5)
C8A	0.0175 (6)	0.0187 (6)	0.0178 (7)	0.0109 (5)	0.0075 (5)	0.0076 (5)
C9	0.0204 (7)	0.0190 (6)	0.0161 (6)	0.0102 (5)	0.0077 (5)	0.0058 (5)
C10	0.0210 (7)	0.0257 (7)	0.0210 (7)	0.0090 (6)	0.0106 (6)	0.0063 (6)
C11	0.0227 (7)	0.0272 (8)	0.0200 (7)	0.0057 (6)	0.0072 (6)	0.0032 (6)
C12	0.0298 (8)	0.0277 (8)	0.0168 (7)	0.0104 (7)	0.0088 (6)	0.0025 (6)
C13	0.0263 (8)	0.0301 (8)	0.0211 (7)	0.0144 (6)	0.0138 (6)	0.0085 (6)
C14	0.0197 (7)	0.0253 (7)	0.0202 (7)	0.0107 (6)	0.0093 (6)	0.0082 (6)
C15	0.0214 (7)	0.0187 (6)	0.0173 (7)	0.0105 (6)	0.0075 (5)	0.0058 (5)
N15	0.0241 (6)	0.0250 (6)	0.0239 (6)	0.0114 (5)	0.0123 (5)	0.0083 (5)
C16	0.0170 (6)	0.0211 (7)	0.0170 (7)	0.0100 (5)	0.0070 (5)	0.0058 (5)
C17	0.0222 (7)	0.0216 (7)	0.0206 (7)	0.0115 (6)	0.0105 (6)	0.0080 (6)
C18	0.0230 (7)	0.0212 (7)	0.0221 (7)	0.0107 (6)	0.0090 (6)	0.0039 (6)
C19	0.0209 (7)	0.0273 (7)	0.0174 (7)	0.0122 (6)	0.0079 (6)	0.0041 (6)
C20	0.0213 (7)	0.0266 (7)	0.0195 (7)	0.0122 (6)	0.0093 (6)	0.0087 (6)
C21	0.0187 (6)	0.0207 (7)	0.0199 (7)	0.0096 (5)	0.0078 (5)	0.0066 (6)
C22	0.0231 (7)	0.0216 (7)	0.0179 (6)	0.0130 (6)	0.0108 (6)	0.0072 (5)
N22	0.0255 (7)	0.0221 (6)	0.0252 (7)	0.0082 (5)	0.0114 (5)	0.0053 (5)
S1	0.01723 (18)	0.01879 (19)	0.01979 (19)	0.00657 (14)	0.00849 (13)	0.00567 (13)
O2	0.0199 (5)	0.0174 (5)	0.0244 (5)	0.0061 (4)	0.0091 (4)	0.0048 (4)
C23	0.0242 (8)	0.0246 (8)	0.0444 (10)	0.0123 (7)	0.0037 (7)	0.0111 (7)
C24	0.0269 (8)	0.0197 (7)	0.0224 (7)	0.0085 (6)	0.0062 (6)	0.0038 (6)

### Geometric parameters (Å, º)

**Table:**

O1—C5	1.2277 (18)	C13—C14	1.384 (2)
N1—C8A	1.3439 (18)	C13—H13	0.9500
N1—C2	1.3792 (18)	C14—H14	0.9500
N1—H1	0.88 (2)	C15—N15	1.152 (2)
C2—N3	1.3178 (18)	C16—C17	1.399 (2)
C2—N2	1.3508 (18)	C16—C21	1.403 (2)
N2—C9	1.4102 (18)	C17—C18	1.388 (2)
N2—H2	0.90 (2)	C17—H17	0.9500
N3—N4	1.4000 (16)	C18—C19	1.392 (2)
N4—C8A	1.3504 (18)	C18—H18	0.9500
N4—C5	1.4003 (18)	C19—C20	1.393 (2)
C5—C6	1.4510 (19)	C19—H19	0.9500
C6—C7	1.403 (2)	C20—C21	1.391 (2)
C6—C15	1.4297 (19)	C20—H20	0.9500
C7—C8	1.4088 (19)	C21—H21	0.9500
C7—C16	1.4829 (19)	C22—N22	1.151 (2)
C8—C8A	1.3988 (19)	S1—O2	1.5253 (10)
C8—C22	1.429 (2)	S1—C24	1.7764 (16)
C9—C10	1.390 (2)	S1—C23	1.7788 (16)
C9—C14	1.394 (2)	C23—H23A	0.9800
C10—C11	1.394 (2)	C23—H23B	0.9800
C10—H10	0.9500	C23—H23C	0.9800
C11—C12	1.388 (2)	C24—H24A	0.9800
C11—H11	0.9500	C24—H24B	0.9800
C12—C13	1.391 (2)	C24—H24C	0.9800
C12—H12	0.9500		
			
C8A—N1—C2	106.74 (12)	C14—C13—H13	119.9
C8A—N1—H1	130.6 (12)	C12—C13—H13	119.9
C2—N1—H1	122.6 (12)	C13—C14—C9	119.96 (14)
N3—C2—N2	128.74 (13)	C13—C14—H14	120.0
N3—C2—N1	112.99 (12)	C9—C14—H14	120.0
N2—C2—N1	118.26 (13)	N15—C15—C6	175.03 (16)
C2—N2—C9	128.42 (13)	C17—C16—C21	119.42 (13)
C2—N2—H2	113.3 (12)	C17—C16—C7	120.65 (13)
C9—N2—H2	118.3 (12)	C21—C16—C7	119.90 (13)
C2—N3—N4	102.08 (11)	C18—C17—C16	120.31 (13)
C8A—N4—N3	112.16 (11)	C18—C17—H17	119.8
C8A—N4—C5	124.35 (12)	C16—C17—H17	119.8
N3—N4—C5	123.34 (11)	C17—C18—C19	120.05 (14)
O1—C5—N4	121.08 (13)	C17—C18—H18	120.0
O1—C5—C6	126.58 (13)	C19—C18—H18	120.0
N4—C5—C6	112.33 (12)	C18—C19—C20	120.12 (14)
C7—C6—C15	122.14 (13)	C18—C19—H19	119.9
C7—C6—C5	124.47 (13)	C20—C19—H19	119.9
C15—C6—C5	113.26 (12)	C21—C20—C19	120.12 (14)
C6—C7—C8	118.34 (13)	C21—C20—H20	119.9
C6—C7—C16	121.23 (13)	C19—C20—H20	119.9
C8—C7—C16	120.43 (13)	C20—C21—C16	119.98 (13)
C8A—C8—C7	117.77 (13)	C20—C21—H21	120.0
C8A—C8—C22	116.44 (12)	C16—C21—H21	120.0
C7—C8—C22	125.79 (13)	N22—C22—C8	173.67 (15)
N1—C8A—N4	106.01 (12)	O2—S1—C24	105.38 (7)
N1—C8A—C8	131.52 (13)	O2—S1—C23	105.07 (7)
N4—C8A—C8	122.47 (13)	C24—S1—C23	99.29 (8)
C10—C9—C14	120.41 (13)	S1—C23—H23A	109.5
C10—C9—N2	123.15 (13)	S1—C23—H23B	109.5
C14—C9—N2	116.43 (13)	H23A—C23—H23B	109.5
C9—C10—C11	118.88 (14)	S1—C23—H23C	109.5
C9—C10—H10	120.6	H23A—C23—H23C	109.5
C11—C10—H10	120.6	H23B—C23—H23C	109.5
C12—C11—C10	121.06 (14)	S1—C24—H24A	109.5
C12—C11—H11	119.5	S1—C24—H24B	109.5
C10—C11—H11	119.5	H24A—C24—H24B	109.5
C11—C12—C13	119.36 (14)	S1—C24—H24C	109.5
C11—C12—H12	120.3	H24A—C24—H24C	109.5
C13—C12—H12	120.3	H24B—C24—H24C	109.5
C14—C13—C12	120.29 (14)		
			
C8A—N1—C2—N3	−1.59 (16)	N3—N4—C8A—C8	179.38 (12)
C8A—N1—C2—N2	178.85 (12)	C5—N4—C8A—C8	−4.9 (2)
N3—C2—N2—C9	1.7 (2)	C7—C8—C8A—N1	−178.46 (13)
N1—C2—N2—C9	−178.83 (13)	C22—C8—C8A—N1	2.0 (2)
N2—C2—N3—N4	−179.43 (14)	C7—C8—C8A—N4	1.4 (2)
N1—C2—N3—N4	1.07 (15)	C22—C8—C8A—N4	−178.22 (13)
C2—N3—N4—C8A	−0.17 (14)	C2—N2—C9—C10	14.8 (2)
C2—N3—N4—C5	−175.92 (12)	C2—N2—C9—C14	−166.01 (14)
C8A—N4—C5—O1	−174.55 (13)	C14—C9—C10—C11	1.3 (2)
N3—N4—C5—O1	0.7 (2)	N2—C9—C10—C11	−179.53 (14)
C8A—N4—C5—C6	6.19 (19)	C9—C10—C11—C12	0.4 (2)
N3—N4—C5—C6	−178.59 (11)	C10—C11—C12—C13	−1.4 (3)
O1—C5—C6—C7	176.10 (14)	C11—C12—C13—C14	0.8 (2)
N4—C5—C6—C7	−4.69 (19)	C12—C13—C14—C9	0.8 (2)
O1—C5—C6—C15	−7.8 (2)	C10—C9—C14—C13	−1.9 (2)
N4—C5—C6—C15	171.43 (11)	N2—C9—C14—C13	178.89 (13)
C15—C6—C7—C8	−173.97 (12)	C6—C7—C16—C17	−134.34 (14)
C5—C6—C7—C8	1.8 (2)	C8—C7—C16—C17	46.20 (19)
C15—C6—C7—C16	6.6 (2)	C6—C7—C16—C21	47.42 (19)
C5—C6—C7—C16	−177.65 (12)	C8—C7—C16—C21	−132.04 (14)
C6—C7—C8—C8A	0.09 (19)	C21—C16—C17—C18	0.1 (2)
C16—C7—C8—C8A	179.57 (12)	C7—C16—C17—C18	−178.18 (13)
C6—C7—C8—C22	179.62 (13)	C16—C17—C18—C19	−0.2 (2)
C16—C7—C8—C22	−0.9 (2)	C17—C18—C19—C20	−0.1 (2)
C2—N1—C8A—N4	1.35 (14)	C18—C19—C20—C21	0.5 (2)
C2—N1—C8A—C8	−178.81 (14)	C19—C20—C21—C16	−0.6 (2)
N3—N4—C8A—N1	−0.77 (15)	C17—C16—C21—C20	0.3 (2)
C5—N4—C8A—N1	174.93 (12)	C7—C16—C21—C20	178.61 (13)

### Hydrogen-bond geometry (Å, º)

**Table:**

*D*—H···*A*	*D*—H	H···*A*	*D*···*A*	*D*—H···*A*
N1—H1···O2	0.88 (2)	1.84 (2)	2.6249 (16)	146.9 (17)
N2—H2···O2	0.90 (2)	2.07 (2)	2.8680 (16)	147.7 (17)
C10—H10···N3	0.95	2.39	2.9956 (19)	121
C23—H23*B*···O1^i^	0.98	2.44	3.166 (2)	131
C24—H24*B*···N22	0.98	2.53	3.474 (2)	162

Symmetry code: (i) *x*+1, *y*+1, *z*.
